# Caring for parents: an evolutionary rationale

**DOI:** 10.1186/s12915-018-0519-2

**Published:** 2018-05-15

**Authors:** J. Garay, S. Számadó, Z. Varga, E. Szathmáry

**Affiliations:** 1 MTA-ELTE Theoretical Biology and Evolutionary Ecology Research Group and Department of Plant Systematics, Ecology and Theoretical Biology, L. Eötvös University, Pázmány P. sétány 1/C, Budapest, H-1117 Hungary; 2 RECENS „Lendület” Research Group, MTA Centre for Social Science, Tóth Kálmán u. 4, Budapest, H-1097 Hungary; 3 Department of Mathematics, Szent István University, Páter K. u. 1, Gödöllő, H-2103 Hungary; 4 Parmenides Center for the Conceptual Foundations of Science, Kirchplatz 1, 82049 Pullach/Munich, Germany; 5 MTA Centre for Ecological Research, Evolutionary Systems Research Group, Klebelsberg Kuno utca 3, Tihany, 8237 Hungary

**Keywords:** 5th commandment, Intra-familiar resource transfer, Kin demography, Menopause, Grandmother, Grandchild

## Abstract

**Background:**

The evolutionary roots of human moral behavior are a key precondition to understanding human nature. Investigations usually start with a social dilemma and end up with a norm that can provide some insight into the origin of morality. We take the opposite direction by investigating whether the cultural norm that promotes helping parents and which is respected in different variants across cultures and is codified in several religions can spread through Darwinian competition.

**Results:**

We show with a novel demographic model that the biological rule “During your reproductive period, give some of your resources to your post-fertile parents” will spread even if the cost of support given to post-fertile grandmothers considerably decreases the demographic parameters of fertile parents but radically increases the survival rate of grandchildren. The teaching of vital cultural content is likely to have been critical in making grandparental service valuable. We name this the Fifth Rule, after the Fifth Commandment that codifies such behaviors in Christianity.

**Conclusions:**

Selection for such behavior may have produced an innate moral tendency to honor parents even in situations, such as those experienced today, when the quantitative conditions would not necessarily favor the maintenance of this trait.

**Electronic supplementary material:**

The online version of this article (10.1186/s12915-018-0519-2) contains supplementary material, which is available to authorized users.

## Background

Darwin [1] raised the possibility that morality has an evolutionary origin. Several models rooted in evolutionary theory shed light on some basic moral issues [2–5]. In contrast, we start with a moral commandment, and investigate whether a phenotype corresponding to this moral commandment wins in a Darwinian struggle for existence, like the investigation of the conditions under which spiteful behavior will die out [6]. Here we investigate the cultural norm that promotes helping parents. We refer to this norm as the Fifth Commandment (see the supplementary information in Additional file 1). This norm has obvious links to biology, and variants of it can also be found in various cultures and religions ranging from those from the East to those from the West (Additional file 1). There is widespread evidence not just for the existence of such a norm but also for the actual support given to parents as well. The form of this support varies across cultures (emotional, instrumental, financial, etc.) and can be a function of other factors, such as the health of elderly parents. This kind of help is readily observed in different cultures: eastern, western [7–11] as well as hunter-gatherer societies [12]. For example, in !Kung hunter-gather community, “old people are highly valued and respected” ([12] p. 78). Moreover, elderly people in a family receive help and this help is key for their high life expectancy: “The death of a spouse and the lack of children or other close relatives to provide care may make it unlikely for a person to survive into old age” ([12] p. 84). Based on the above, we introduce the so-called Fifth Rule, which is a translation of the Fifth Commandment into biological terms and is inherent in the above interpretations: “During your reproductive period, give some of your resources to your post-fertile parents.” Investigating the dynamics of this norm may shed light on the evolutionary roots of religion also [13].

During the standard life history of humans, infants grow to become parents who age into grandparents. Thus, longevities permitting, respect and help for parents become targeted at the grandparents of one’s children. This truism has important consequences for the possible spread of such a behavioral trait. Behaviors can be inherited, by either genetic or cultural transmission. This inheritance assumption immediately implies that if the support given to grandparents spreads by Darwinian selection, then that ensures longer life for the parents, as their children will inherit their behavior. Like classical evolutionary game theory, we will not consider the genetic or potential cultural background of the behavior [14]. We assume that this behavior evolved when the potential for horizontal cultural transfer was negligible due to the low population density of humans [15]; thus, the success of genetically determined behaviors and the success of culturally determined behaviors were tightly linked. An adaptive phenotype will outperform its rivals on a Darwinian selection time scale, regardless of whether it is coded genetically or culturally. Here, Darwinian fitness is the average growth rate of a phenotype.

The establishment of a post-fertile period is critical for our case. Several hypotheses deal with the origin of the menopause.

Shanley and Kirkwood [16] investigate two alternative theories that might explain the origin of the menopause. The first, called the altriciality hypothesis, observes that maternal mortality increases with age. It implies a trade-off between rearing existing, still altricial children and giving birth to a new one. The second is the mother hypothesis, which states that a post-fertile grandmother can help her fertile daughter [17]. They found that neither of these ideas alone is sufficient to explain the evolution of menopause under a realistic range of life-history parameters; however, a combined model can explain it [16, 18]. Their conclusion is corroborated by other studies, both on altriciality [19] as well as on kin selection [20].

**Fig. 1:**
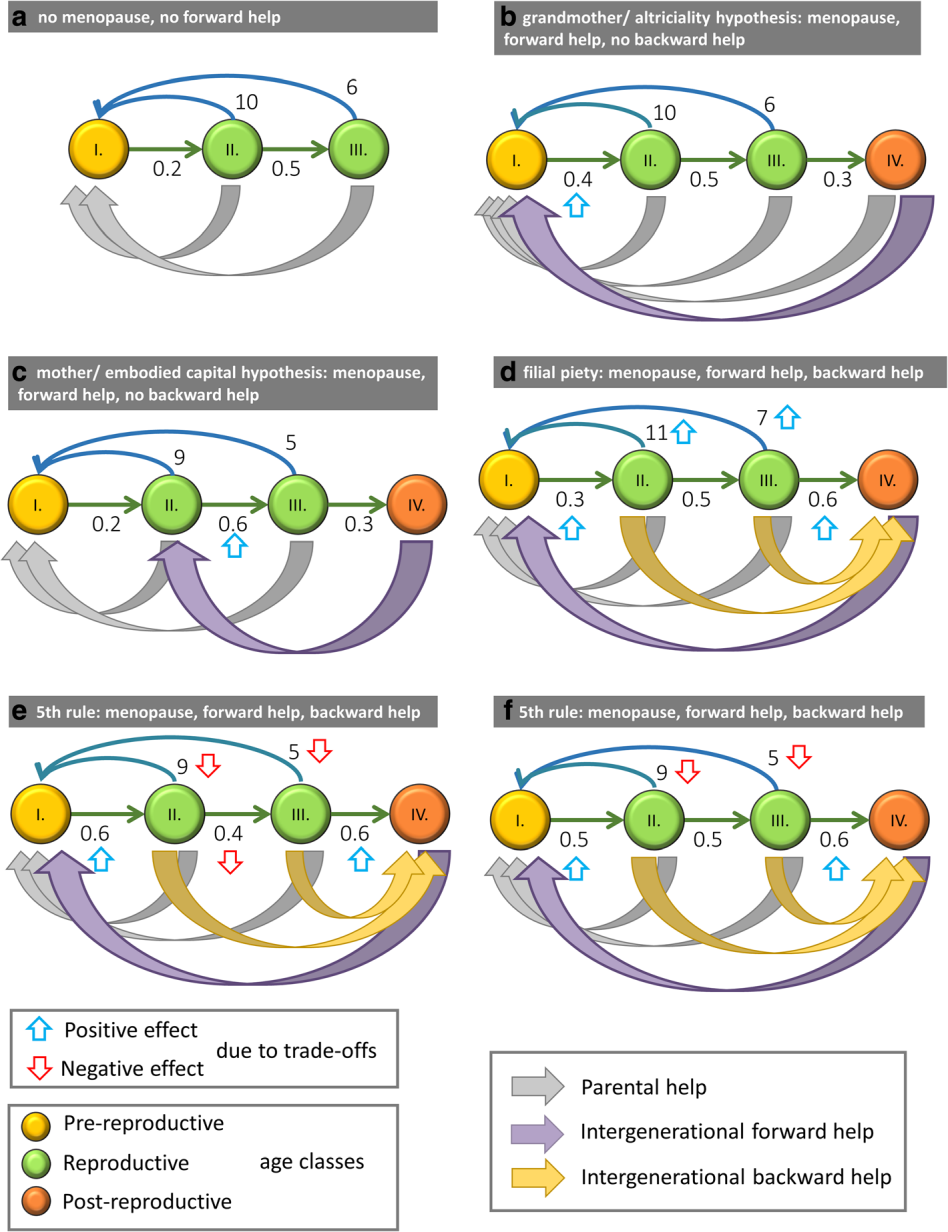
The different theories. Grey arrows denote parental help, purple arrows denote forward help from grandmothers to a grandchild, and finally, yellow arrows denote the backward transfer of resources from parents to grandparents. Upward blue and downward red arrows denote the positive and negative effects from the trade-offs, respectively. **a** Standard life-history model with no menopause, no forward help, and no backward help. **b** Grandmother (purple arrow from VI to I) and altriciality (grey arrow from VI to I) hypotheses. The menopause has evolved and there is no backward help from parents to grandmothers. **c** Mother and embodied capital hypotheses. The menopause has evolved and there is no backward help from parents to grandmothers. **d** Filial piety. The menopause has evolved, there is a synergistic division of labor with backward help from parents to grandmothers, and there are no trade-offs; **e** Fifth rule. The menopause has evolved, there is backward help from parents to grandmothers, and there is a three-way trade-off for the parents between survival, fecundity, and helping their grandmothers. **f** Fifth Rule. The menopause has evolved, there is backward help from parents to grandmothers, and there is a two-way trade-off for the parents between fecundity and helping their grandmothers

According to the grandmother hypothesis [21–26], the advantage of the post-fertile stage is that grandmothers enhance the survival of their grandchildren [22, 26, 27], by increasing either the survival rate or the fecundity of the latter [28, 29]. A third hypothesis is the embodied capital model, which emphasizes that the intergenerational transfer (IT) of skills, knowledge, and social ability needs time, and both grandmothers and grandfathers could help in the training of their grandchildren [30]. The skills and knowledge attained during childhood can increase the survival rate and fecundity for the whole adult life of the grandchildren; see Fig. 1a–c for a comparison of these alternatives. These three hypotheses do not necessary exclude each other, since the care for pre-fertile individuals includes breastfeeding, transport, feeding, and protection as well as affection and education [27, 31, 32].

All these hypotheses are aimed at explaining the evolutionary advantage of the long post-fertile life period of *Homo sapiens*. However, none of them assumes that there is a transfer of resources from the parents to the grandparents, thus none of them investigates the trade-off between parental reproduction or survival and the support given to grandparents. The central question is this: Will the support given to post-fertile grandmothers spread even if there is a trade-off between this support and either the fecundity or the survival rate of fertile parents?

Cyrus & Lee [3] investigated the evolution of IT from parents to grandparents in the framework of a cooperative game. They showed that filial piety can evolve through the division of labor. A fertile female transfers some of her energy to her mother, enabling the latter to redirect her efforts from inefficient foraging to the care of her grandchildren, allowing the fertile female to forage, doing so with higher efficiency than her mother. In other words, this model describes a synergistic situation where everyone does the task she is the most efficient at. However, the authors do not consider the trade-off we wish to investigate (see Fig. 1d).

We strongly concur with the statement that “Even to demonstrate, for example, that post reproductive women result in a reduction in grandchild mortality *does not establish that menopause is adaptive unless it can be demonstrated that overall fitness is actually enhanced*.” [18] (p. 27, their emphasis). In establishing the selective advantage of caring for grandmothers, we consider the effect of the overall fitness of the family.

Since in our problem, pre-fertile, fertile, and post-fertile individuals live together in a family, we have to consider a kin demographic selection model [3, 33, 34], in which the survival and the fecundity parameters depend on the costs and benefits of intra-familiar supports. After setting up the model, we investigate whether the Fifth Rule (as a biological distillation of the Fifth Commandment; see Additional file 1) wins in a Darwinian struggle for existence. Finally, we discuss our results.

## Methods

We consider a Leslie matrix model (see Table 1 for notation). Our model strictly follows the Darwinian view: fitness is determined by fecundity and the survival rate. The fecundity of a family is determined by the intergenerational help, which modifies the demographic parameters within the family. Furthermore, the carrying capacity also has an effect on survival. Thus, the survival of an individual depends on intra-familial help and on the survival probability according to the carrying capacity: our model combines these two factors.

**Table 1:** Model notation

Life-history parameters	
*α*	Number of offspring
*ω*_1_	Survival of the first age class (offspring)
*ω*_2_	Survival of the first reproductive class (parents)
*ω*_3_	Survival of the non-reproductive class (grandparents)
Benefit parameters	
*a*_21_	Efficacy of a grandparent’s help on the survival of the offspring
*b*	Effectiveness of backward help, the maximum efficacy of the parents’ help on the grandparent’s survival

**Fig. 2:**
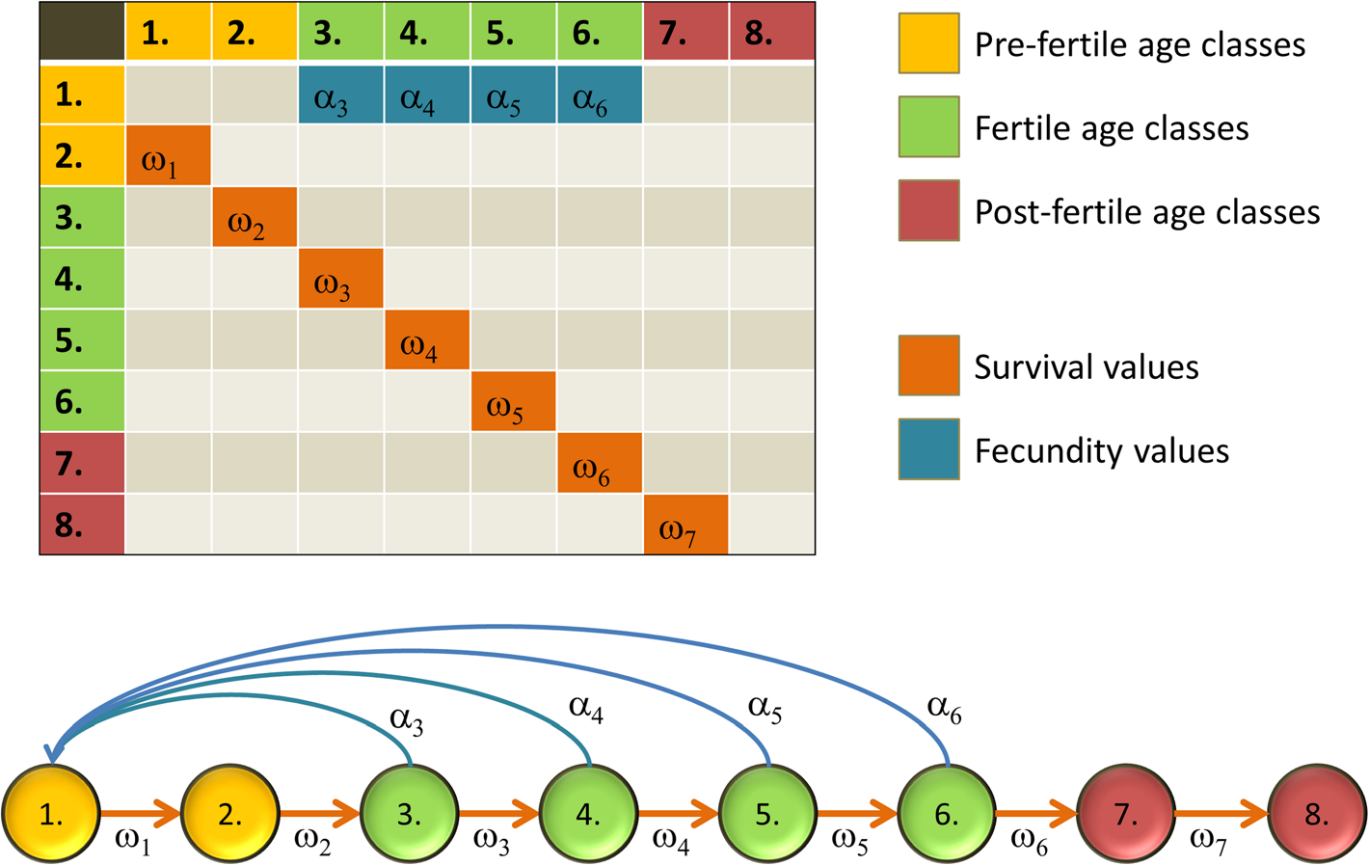
The Leslie matrix. Yellow, green, and red represent the pre-fertile, fertile, and post-fertile age classes, respectively. The fecundities of the reproductive age classes are denoted by the *α*’s and the survival rates of the age classes are represented by the *ω*’s

We consider the following age-structured model with two sub-models. The development of a family is described by the following Leslie matrix, which contains the survival and fecundity parameters of pre-fertile and fertile individuals, and all entries depend on the level of the intra-familiar (backward) help, denoted by *y*:

1 $$ \left(\begin{array}{cccccccc}0& 0& \dots & 0& {\alpha}_{k+1}(y)& \dots & {\alpha}_{K-1}(y)& {\alpha}_K(y)\\ {}{\omega}_1(y)& 0& \dots & 0& 0& \dots & 0& 0\\ {}0& {\omega}_2(y)& \dots & \dots & & .\dots & & \\ {}0& 0& \dots & 0& & .\dots & & \\ {}& & \dots & {\omega}_k(y)& 0& \dots & & \\ {}& & \dots & 0& {\omega}_{k+1}(y)& .\dots & & \\ {}& & \dots & \dots & 0& \dots & 0& \\ {}0& 0& \dots & 0& & \dots & {\omega}_{K-1}(y)& 0\end{array}\right), $$

where *ω*_*i*_(*y*) (*i* = 1, …, *k*) denote the survival rates of children, and *ω*_*j*_(*y*) and *α*_*j*_(*y*) (*j* = *k* + 1, …, *K*) are the survival rate and fecundity of fertile parents, respectively. Figure 2 depicts an example. The product of the age-structured population vector and this Leslie matrix describes the dynamics of the family. The age classes of grandparents will be handled separately, since the development of the family depends on the survival rate of pre-fertile family members and the fecundity of the fertile family members. Formally, *x*_*l*_ = *ω*_*l* − 1_(*y*)*x*_*l* − 1_ where *ω*_*l*_(*y*) (*l* = *K* + 1, …, *H*) are the survival rates of the grandparents, and *x*_*l*_ is the number of grandparents in age class *l.*

In the framework of the Leslie model, it is widely accepted that the Darwinian fitness is the long-term growth rate of the phenotype (i.e., the dominant positive eigenvalue of the Leslie matrix). Surprisingly, we could not find in the literature a Darwinian explanation of this. Below, adapting our recent reasoning from [35], we propose a strictly Darwinian rationale to show that the long-term growth rate is maximized by natural selection (see Additional file 1, Section 1 for details).

What is the effect of the Fifth Rule on the entries of the above Leslie matrix? For the simplest mathematical formulation, we assume that the cost of supporting grandmothers does not depend on the age class of either parents or grandmothers. Let *y* ∈ [0, 1] be the cost spent on supporting grandparents. If grandmothers help in child care, the survival rates of children *ω*_*i*_ increase with increasing *y*, and based on the grandmother and the mother hypotheses, *ω*_*j*_ decreases and *α*_*j*_ increases with increasing *y*, where *ω*_*i*_(*y*) (*i* = 1, …, *k*) denote the survival rates of children, and *ω*_*j*_(*y*) and *α*_*j*_(*y*) (*j* = *k* + 1, …, *K*) are the survival rate and fecundity of fertile parents, respectively.

Since there is a difference in intra-familiar support between families, the Leslie matrices of different family types are different. What kind of intra-familiar support ensures the highest long-term growth rate for the family? For simplicity. we denote help from grandmothers to children as forward help and help from parents to grandparents as backward help. (See Fig. 1 for a comparison of the different models.) Under well-known conditions (fulfilled in our case), the unique positive eigenvalue of the Leslie matrix is the long-term growth rate of the family; thus, we consider this eigenvalue to be the fitness [36, 37]. Formally, the fitness *λ*(*y*) is the unique positive eigenvalue of the *y*-dependent Leslie matrix; hence, other things being equal, families in which grandmothers are helped are competitively superior to those without this behavior.

## Results

### Grandmother hypothesis

**Fig. 3:**
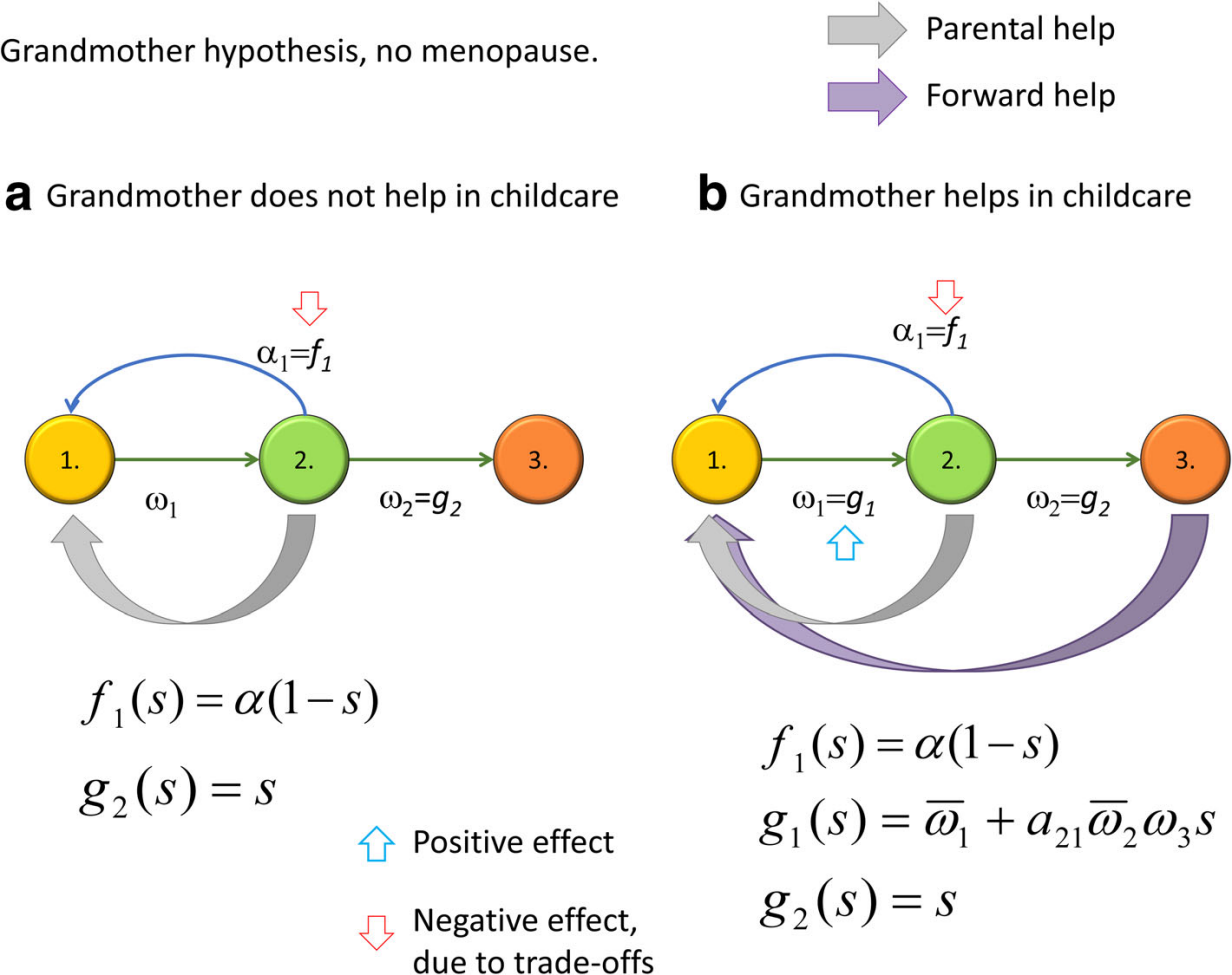
Grandmother hypothesis without (**a**) and with (**b**) child care. Green arrows denote parental help and purple arrows denote forward help from grandparents to a grandchild

Consider the case when fertile individuals do not support grandmothers (see Fig. 1e for a general depiction of the idea). We consider the following two cases (see Additional file 1 and Fig. 3 for details):

(i) If grandmothers do not help in child care but survival linearly reduces fecundity of the fertile age class, then the optimal strategy is not to spend on one’s own survival to post-fertile age.

(ii) If grandmothers help in child care then the menopause is evolutionarily successful if the effect of grandchild care (${a}_{21}{\overline{\omega}}_2{\omega}_3$) on a grandchild’s survival (${\overline{\omega}}_1$) is greater than the survival rate without this care, i.e., ${a}_{21}{\overline{\omega}}_2{\omega}_3>{\overline{\omega}}_1$ (see Table 1 for notation, where ${\overline{\omega}}_{\ast }$ denotes averages).

In summary, the grandmother hypothesis concerns the way a female of reproductive age allocates her resources between her own survival and her own fecundity. Note that we have adopted the hypothesis that the cost spent on living to the post-fertile age reduces fecundity. Without this trade-off, living to the post-fertile age is a neutral property in the first case and a benefit in the second case.

### The Fifth Rule

**Fig. 4:**
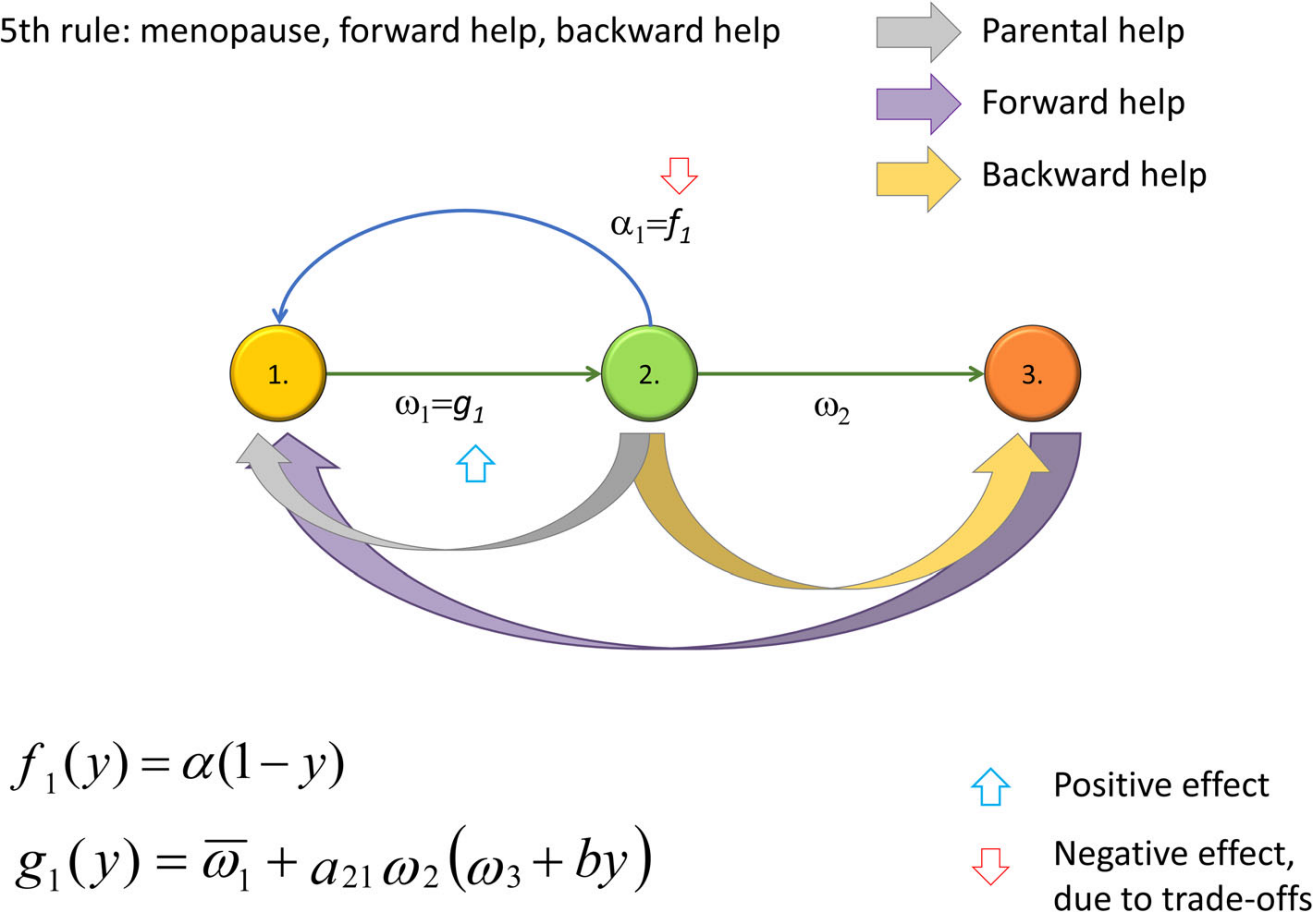
The Fifth Rule. There is forward help in child care and backward help given to grandparents. Green arrows denote parental help, purple arrows denote forward help from grandparents to a grandchild, and finally, red arrows denote the backward transfer of resources from parents to grandparents

The Fifth Rule requires us to support our elderly (see Fig. 1f for a general depiction of the idea), which may occur when the menopause has already become evolutionarily fixed (see Additional file 1 and Fig. 4 for details). We show (Additional file 1) that the Fifth Rule (backward help) evolves when${a}_{21}{\omega}_2\left(b-{\omega}_3\right)>{\overline{\omega}}_1$. This condition is satisfied if, for example, the efficiency of the support given to post-fertile parents is sufficiently large compared to the basic post-fertile survival rate (if the latter were high, than grandmothers would be around even if they were not helped).

Of course, the coevolution of two traits—a long life after the menopause and an effective Fifth Rule—is also possible. The analytical study presented in Additional file 1 is based on two conditions. First, the development of the Fifth Rule is conditional on the existence of the menopause, since one can help a grandmother only if she is alive. (Based on this conditionality, in Additional file 1, we suppose that traits *s* and *y* determine both the increase of the grandmother’s survival probability and the decrease of fecundity in multiplicative form.) Consequently, the rarity of an effective Fifth Rule is hardly surprising. Second, if a fertile mother were to give all her resources to help the survival of her mother, her fecundity would drop to zero. In Additional file 1, in terms of a fitness landscape, we show that, if the fitness*λ*(*s*, *y*) has a strict global maximum (*s*^∗^, *y*^∗^) (e.g., in our case, if *λ*(*s*, *y*)) were strictly concave), then there exists a unique evolutionarily optimal behavior (*s*^∗^, *y*^∗^); hence, the species evolves into this state.

The Fifth Rule will spread if the cost of the support given to post-fertile grandmothers slightly decreases the demographic parameters of fertile parents, but sufficiently increases the survival rate of grandchildren. However, in general, there is a threshold over which support given to grandmothers has no evolutionary advantage. If the cost of support given to post-fertile grandmothers only decreases the demographic parameters of the family but offers no increase in the survival rate of the grandchildren, then the Fifth Rule has no evolutionary advantage. The mother hypothesis and embodied capital model should imply that grandmothers increase the survival rate of their children and that of grandchildren during their lives. Thus, if these ideas also work in human evolution, then it is even easier for the Fifth Rule to evolve (see Additional file 1).

**Fig. 5:**
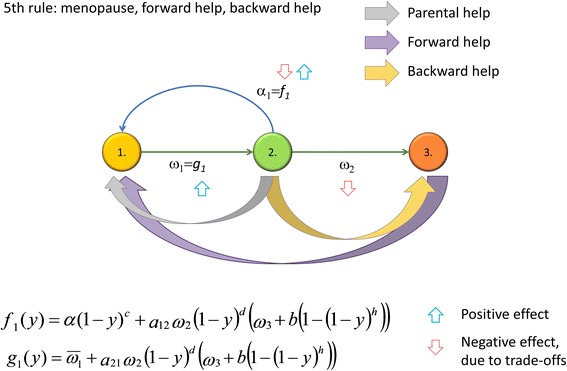
General two-age-class model. Green arrows denote parental help, purple arrows denote forward help from grandparents to a grandchild, and finally, red arrows denote the backward transfer of resources from parents to grandparents

**Fig. 6:**
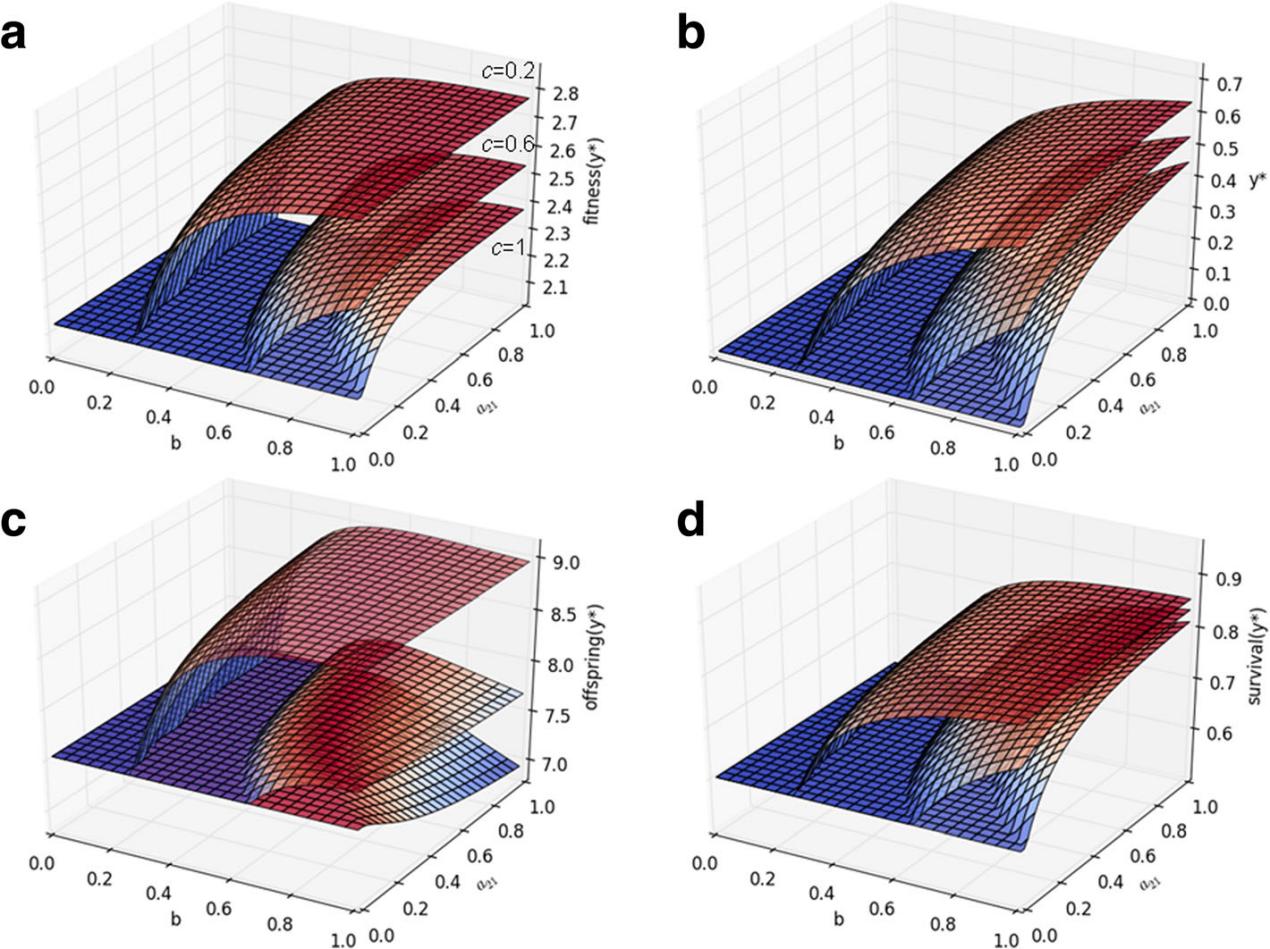
Numerical example for a 2 × 2 Leslie matrix (see Additional file 1 for details). **a** Maximum family long-term growth rate (fitness), **b** optimal level of backward help (y*), **c** average number of offspring at y*, and **d** offspring survival at y* all as a function of *b* (effectiveness of backward help) and *a*_21_ (effectiveness of forward help on offspring survival). Parameters: *α*_2_ = 6, *ω*_1_ = 0.45, *ω*_2_ = 0.62, *ω*_3_ = 0.25, *d* = 0.3, *h* = 1, *c* = 0.2, 0.6, 1, and *a*_12_ = 10

To investigate the effects of different cost–benefit parameters on the evolvability of IT, we constructed a general example, which we analyzed numerically (see Additional file 1 and Fig. 5 for details). Our conclusions from the model are as follows. IT evolves most readily when grandparental help increases both the survival and the number of offspring [22, 26, 27] (Fig. 6, Additional file 1: Figures S1–S3). Linear cost and benefit functions do not favor the evolution of IT (Additional file 1: Figures S1, S4, and S6). Conversely, convex benefit and concave cost functions promote the evolution of IT (Additional file 1: Figures S2, S3, S5, and S7). It is possible to find cost parameters (*c*, *d*) for which IT evolves even if the efficacy of parental transfer and grandparental help (*a*_21_ and *b*, respectively) is low (Additional file 1: Figures S2 and S3). Conversely, it is possible to find (high) *a*_21_ and *b* parameters for which IT evolves even if it imposes a high cost on the survival of the parents or on the number of offspring (*d* and *c*, respectively, see Additional file 1: Figures S1 and S2).

Since we are dealing with family issues, the natural conceptual framework is that of kin selection. Although some works incorporate demography into inclusive fitness analyses, and also consider intergenerational resource transfers (e.g., Johnstone & Cant 2010), our model also involves, in addition, an unusual loop from a parent to a grandparent to a grandchild. There are different contributions to a female’s fitness from the three stages of her life history, as girl, mother, and grandmother. Our demographic model can account for these complications in a straightforward manner. Our analysis applies to grandfathers as well, provided the menopause in grandmothers constrains the realized fertility of the former in a similar way.

## Discussion

We have shown that the biological version of the Fifth commandment called the “Fifth rule” can spread by means of natural selection under fairly general conditions. Our argument presented in the paper focuses on grandmothers. However, helping elderly parents is not constrained to females. Does our argument hold for males as well? To understand the argument, it is important to differentiate between fertility or loss of it (i.e., the male menopause or andropause) and reproductive success in general. It is well established that testosterone levels decrease with age in men [38, 39] and that this decrease is very often paralleled with depression, nervousness, decreased libido, erectile dysfunction, poor concentration, and memory [40]. The collection of these symptoms is called the male menopause [40]. Both the usefulness of the term and the idea that these symptoms can be traced back to one cause are hotly debated [40–43]. It is clear that the decline in fertility is not as sharp and not as general as in females [40, 41]. A recent study concludes that “the existence of the clinical and biochemical syndrome known as [late-onset hypogonadism] has been confirmed, but its incidence appears to be notably lower than originally estimated” [42]. However, we think that the existence of the male menopause (a sharp decline of fertility) is not crucial to our argument. Even if a grandfather’s fertility remains unchanged, his reproductive success is expected to drop for several reasons as fertility is just one (necessary) component of reproductive success:

(i) The menopause of the grandmother denies the grandfather the most obvious reproductive opportunity.
(ii) It is probable that grandfathers will not be as successful as young males in the competition for younger females (i.e., they will enjoy fewer mating opportunities).
(iii) Even if the grandfather is successful in mating, the child might not be counted as his child, thus it requires no further resources from the grandfather, which in turn implies the grandfather will be free to provide care for his (official) grandchildren.
(iv) Older men are also more prone to cuckoldry [44]. Bribiescas [44] concludes in a review on male reproductive senescence that “while the physiological potential for fathering offspring remains intact well into the later stages of a male’s life, somatic degradation that results in a decline in attractiveness, sexual motivation, energy availability, and a compromised ability to acquire resources may indeed result in a form of male reproductive senescence that severely restricts male fitness at older ages” (p. 138). Overall, most elderly males will be forced out of reproduction, either because of the loss of fertility or because of the loss of mating opportunities; hence, our arguments as presented for grandmothers apply to grandfathers as well.

The useful contribution of the grandfather to his grandchildren (especially grandsons) might manifest itself later in childhood, due to, among others, the teaching of survival practices (e.g., successful hunting). For example, amongst the hunter-gatherers of the eastern boreal forests of North America “older males acquire, harbor, and are reservoirs for enormous scales of spatial information on both resources and mobility” ([45],abstract).

## Conclusions

We demonstrate that an essential part of the Fifth Commandment (supporting the elderly) can confer a selective advantage under the right conditions; hence some kind of evolutionary moral sense might be genetically endowed. This holds if grandparents have a positive effect on the growth rate of their family. However, this is not necessarily true nowadays [46]. It is very well possible that this support is rooted in past human evolution. The Darwinian success of the Fifth Rule cannot completely explain the present-day Fifth Commandment. Human moral rules, although rooted in Darwinian evolution, are more than what that theory supports. It seems that the main difference is that moral commandments are unconditional rules, while in Darwinian evolution there must be a selective condition determining whether a behavior is adaptive or not.

## Additional file

Additional file 1: Supporting material. Supplementary discussion, supplementary methods, **Figures. S1–S7**, and **Tables. S1 and S2** (PDF 1152 kb)
