# Supplementary material for: Caring for parents: an evolutionary rationale
Source: BMC Biol. 2018 May 15;16:53. doi: 10.1186/s12915-018-0519-2 (PMC5953408; doi:10.1186/s12915-018-0519-2)
Supplement: Supplementary file 1 — Supporting material. Supplementary discussion, supplementary methods, Figures. S1–S7, and Tables. S1 and S2 (PDF 1152 kb) [file 12915_2018_519_MOESM1_ESM.pdf]

Caring for parents: an evolutionary rationale

Supplementary Information

J. Garay[1,5], S. Számadó[2,5], Z. Varga[3], E. Szathmáry[4,5]*

April 3, 2018

[1] MTA-ELTE Theoretical Biology and Evolutionary Ecology Research Group and Department of Plant Systematics, Ecology and Theoretical Biology, L. Eötvös University, Pázmány P. sétány 1/C, H-1117 Budapest, Hungary.

[2] RECENS „Lendület" Research Group, MTA Centre for Social Science. H-1097, Budapest, Tóth Kálmán u. 4.

[3] Department of Mathematics, Szent István University, Páter K. u. 1. H-2103, Gödöllő, Hungary.

[4] Parmenides Center for the Conceptual Foundations of Science, Kirchplatz 1, 82049 Pullach/Munich.

[5]MTA Centre for Ecological Research, Evolutionary Systems Research Group., Klebelsberg Kuno utca 3, Tihany 8237, Hungary.

*Correspondence to: Eörs Szathmáry, email: szathmary.eors@gmail.com.

Supplementary Discussion

Cultural analogues of the Fifth Commandment

Cultural norms that promote the help of the parents are widespread in both western and eastern culture. The Fifth Commandment (of the Hebrew and protestant Bible, the Fourth one, according to the catholic numbering) states: "*Honor your father and your mother, that your days may be long in the land that the LORD your God is giving you.*" (Exodus 20:12)

From the interpretations of this commandment by the western churches we recall the following: Sefer Ha-chinukh (mitzva 33) elaborates: "*A person should realize that his father and mother are the cause of his existence in this world; therefore it is appropriate that he render them all the honor and do them all the service he can*". St. Thomas Aquinas wrote: "*Since we receive nourishment from our parents in our childhood, we must support them in their old age*." Martin Luther said: "*For he who knows how to regard them in his heart will not allow them to suffer want or hunger, but will place them above him and at his side, and will share with them whatever he has and possesses*" (Luther, M. p. 29).

We also note that in China, to take care of elderly parents is also a moral rule: e.g. Confucius declared: "*In serving his parents, a filial son reveres them in daily life; he makes them happy while he nourishes them; he takes anxious care of them in sickness ...*" (*26*)

Based on the above, we introduce the so-called Fifth Rule, which is a translation of the Fifth Commandment into biological terms and is inherent in the above interpretations: "During your reproductive period, give away from your resources to your post-fertile parents."

Supplementary Methods

In the following we investigate a biological model for intra-familiar help, give some general results on it, and finally, we investigate the simplest model with numerical examples.

1. The survival at the carrying capacity

Here we propose a strictly Darwinian reasoning to see that the long-term growth rate is maximized by natural selection: the number of offspring, in general, is much higher than the carrying capacity, so only a part of the offspring and adults will survive. Let us consider random survival, assuming that the survival probabilities of individuals do not depend on phenotypes (in our case intergenerational help) and on the age of individuals. (Observe that this assumption gives some advantage to the families in which the intergenerational help is less.)

Now let us consider two phenotypes A and B with respective long-term growth rates (i.e. positive eigenvalues of the corresponding Leslie matrices) $\lambda_A, \lambda_B > 1$ with $\lambda_A > \lambda_B$. To see the asymptotic frequency of phenotype B, we suppose that phenotypes A and B start from respective initial densities $x(0)$ and $z(0)$. According to the original Darwinian view, we need some density dependent selection to keep the total density of these two phenotypes at the carrying capacity. Since in the considered selection situation there is no interaction between the phenotypes and we assume that the phenotypes differ only in the demographic parameters, thus we can suppose there is a uniform survival process, i.e. the survival rate corresponding to the carrying capacity is the same for all individuals. Now the question arises which phenotype will win in the struggle for existence on the long selection time scale?

Let us suppose that phenotypes A and B develop according to Leslie models having the respective population vectors $x(t)$, $z(t)$, and matrices L_A, L_B, total densities

$\|x(t)\| = \sum_i x_i(t),\ \|z(t)\| = \sum_i z_i(t)$. Then the relative frequency of phenotype B tends to zero, as it is shown below:

Indeed, let us suppose that the subpopulations start from initial states $x(0)$ and $z(0)$, respectively, and the time unit is chosen in such a way that in unit time the total density of the system always exceeds the carrying capacity *K*, in particular

$$\|L_{\mathrm{A}}x(0)\| + \|L_{\mathrm{B}}z(0)\| > K. \qquad \text{(SI 2)}$$

Now, by the selection the total density of the system is reduced to *K* proportionally:

$$x(1) = \frac{K}{\|L_{\mathrm{A}}x(0)\| + \|L_{\mathrm{B}}z(0)\|} L_1 x(0), \qquad \text{(SI 3)}$$

$$z(1) = \frac{K}{\|L_{\mathrm{A}}x(0)\| + \|L_{\mathrm{B}}z(0)\|} L_{\mathrm{B}} z(0). \qquad \text{(SI 4)}$$

Indeed, obviously $\|x(1)\| + \|z(1)\| = K$.

We emphasize that in this model we consider the "intrinsic" survival (described by the Leslie matrices) and the survival under selection independently. However, this model can be formally considered as a particular Leslie-type model depending on the total density of the system, where each demographic parameter in the Leslie matrices L_{A} and L_{B} is multiplied by $\dfrac{K}{\|L_{\mathrm{A}}x(0)\| + \|L_{\mathrm{B}}z(0)\|}$.

Similarly, for all *t*= 1, 2, 3,… we get our kin demographic selection model for two different phenotypes:

$$\begin{aligned} x(t+1) &= \frac{K}{\|L_{\mathrm{A}}x(t)\| + \|L_{\mathrm{B}}z(t)\|} L_{\mathrm{A}} x(t) \\ z(t+1) &= \frac{K}{\|L_{\mathrm{A}}x(t)\| + \|L_{\mathrm{B}}z(t)\|} L_{\mathrm{B}} z(t) \end{aligned}. \qquad \text{(SI 5)}$$

Now, for the proportion of phenotype B we obtain

$$\frac{\|z(t)\|}{\|x(t)\|+\|z(t)\|}=\frac{1}{\frac{\|x(t)\|}{\|z(t)\|}+1}\ . \quad \text{(SI 6)}$$

Here

$$\frac{\|x(t)\|}{\|z(t)\|}=\left(\frac{\lambda_A}{\lambda_B}\right)^t \frac{\frac{\|x(t)\|}{\lambda_A^t}}{\frac{\|z(t)\|}{\lambda_B^t}}. \qquad \text{(SI 7)}$$

Since we can suppose that in both phenotypes the last two fecundities are positive, so the Perron-Frobenius theorem (see e.g. 28) implies that both $\frac{\|x(t)\|}{\lambda_A^t}$ and $\frac{\|z(t)\|}{\lambda_B^t}$ tend to finite positive limits as $t \to \infty$. In fact, the Leslie matrices can be cut at the last fertile age class, apply the Perron-Frobenius theorem to these matrices, and then the convergence can be extended to the post fertile age groups by simple survival Therefore, $\lim_{t\to\infty}\frac{\|x(t)\|}{\|z(t)\|}=\infty$, implying

$$\lim_{t\to\infty}\frac{\|z(t)\|}{\|x(t)\|+\|z(t)\|}=0\,. \text{(SI 8)}$$

Thus if $\lambda_A > \lambda_B$, then the relative frequency of phenotype B tends to zero as t tends to infinity. Observe that in our model, the fecundity of a phenotype is determined by a phenotype-dependent Leslie matrix, and the survival rates corresponding to the carrying capacity of different phenotypes are the same, so the long-term growth rate of a phenotype determines the fitness.

2. The general results

Consider the general $K \times K$ Leslie matrix, where the entries depend on the cost y spent to grandparent support. Under the grandmother hypothesis, the grandmother support decreases the fecundity and survival rate of fertile parents, but increases the survival rate of the grandmother, who therefore increases the survival rate of pre-fertile grandchildren. Then the characteristic equation is

$$\lambda^K - \alpha_{k+1}(y)\prod_{i=1}^{k}\omega_i(y)\lambda^{K-(k+1)} - \alpha_{k+2}(y)\prod_{i=1}^{k+1}\omega_i(y)\lambda^{K-(k+2)} - .... - \alpha_K(y)\prod_{i=1}^{K-1}\omega_i(y) = 0, \qquad \text{(SI 9)}$$

and its unique positive root is obtained as the root of equation

$$q(\lambda) = \frac{\alpha_{k+1}(y)\prod_{i=1}^{k}\omega_i(y)}{\lambda^{k+1}} + \frac{\alpha_{k+2}(y)\prod_{i=1}^{k+1}\omega_i(y)}{\lambda^{k+2}} + ... + \frac{\alpha_K(y)\prod_{i=1}^{K-1}\omega_i(y)}{\lambda^K} = 1. \qquad \text{(SI 10)}$$

It is easy to see, that if any of the numerators (i.e. the average numbers of offspring produced by an individual of the corresponding age classes) in these fractions is changed to a greater one, then the curve of the 'hyperbolic' function q shifts upwards, implying that the positive solution λ_* of this equation also will be greater. Therefore, if in a population where within the families grandparents are not supported, a new type emerges which supports grandparents, and all mentioned numerators increase, then Fifth Rule as behaviour type will propagate. If all these numerators decrease then this type will die out. Those mathematical cases when some of the numerators increase, others decrease, would need further mathematical discussions.

Observe that equation $q(\lambda) = 1$ can be written as

$$\prod_{i=1}^{k}\omega_i(y)\left(\frac{\alpha_{k+1}(y)}{\lambda^{k+1}} + \frac{\alpha_{k+2}(y)\omega_{k+1}(y)}{\lambda^{k+2}} + ... + \frac{\alpha_K(y)\prod_{j=k+1}^{K-1}\omega_j(y)}{\lambda^K}\right) = 1. \qquad \text{(SI 11)}$$

Here factor $\prod_{i=1}^{k}\omega_i(y)$ measures how much child care by grandmothers increases the survival of the children. Roughly speaking, factor

$$\frac{\alpha_{k+1}(y)}{\lambda^{k+1}}+\frac{\alpha_{k+2}(y)\omega_{k+1}(y)}{\lambda^{k+2}}+....+\frac{\alpha_K(y)\prod_{j=k+1}^{K-1}\omega_j(y)}{\lambda^K} \qquad \text{(SI 12)}$$

measures, in an implicit way, to what extent the support to grandparents by the fertile age class decreases their own fecundity and survival rates. In this sense, the predictions of our model are in harmony with the cost-benefit approach saying that a trait will propagate if it eventually increases the fitness.

Now the question arises how the demographic parameters may depend on y. The following assumptions are at hand: 1. The survival rate of grandparents is a saturation function of y strictly increasing at the beginning, and remains constant after. 2. Based on the grandmother hypothesis, the survival rate of grandchildren strictly increases with the survival rate of grandparents (which on its term depend on y). The grandmother hypothesis is the worst case when two trade-offs may exist. 3. The parents' fecundity entries of the Leslie matrix ($\alpha_{k+1},...,\alpha_K$) are strictly decreasing functions of y. 4. The survival rates of parents ($\omega_{k+1},...,\omega_K$) are strictly decreasing functions of y.

These assumptions allow the Fifth Rule to win or lose the struggle for existence, depending on whether the long-term growth rate of the family increases or decreases.

Under assumption 1 there is a threshold for the support to grandparents, above which the survival of grandparents does not increase, and therefore the survival of grandchildren

either, but the fecundity and/or the survival of fertile parents still decrease. Over this threshold, the support to grandparents has no evolutionary advantage.

Finally, we remark that the above reasoning can be applied not only to the grandmother hypothesis, since either the mother hypothesis or the embodied capital model alone can ensure the support to grandparents. For example, if any of the above two hypotheses implies the increase of at least one of the numerators in (3), while the rest of the numerators do not decrease, then the dominant eigenvalue, i.e. the asymptotic growth rate will increase. Of course, if in addition to the fact that the grandmother increases the survival of her grandchildren and the survival and fertility of her daughter, the hypothesis of the embodied capital model also holds (the grandmother also increases the adult age survival and fertility of her grandchildren), then not only $\prod_{i=1}^{k} \omega_i(y)$ but also factor (4) can synergically increase the asymptotic growth rate of the family.

Finally, we note that the "altriciality" hypothesis can also be handled in terms of a linear model with a matrix structured differently from the Leslie matrices (since the survivals of children also depend on the age of their mothers). Thus, only a generalization our model could deal with the development of menopause based on altriciality. In our opinion, our Fifth Rule may be derived on the bases of "altriciality" hypothesis, but in such a future model the formation of multi-generation families should also be included, since "altriciality" hypothesis itself does not need the convivence of several generations.

3. Two-age-class model

For a deeper insight, in this simplest case, we will calculate first when the menopause can evolve, second, when the Fifth Rule is evolutionary successful, third, using numerical examples we demonstrate that convex benefit and concave cost functions promote the evolution of intra-familiar help.

Consider the simplest case with one child age class and one fertile age class. Then the Leslie matrix is

$$\begin{pmatrix} 0 & \alpha_0 \\ \omega_1 & 0 \end{pmatrix},$$

where ω_1 is the survival rate of children and α_0 is the fecundity of fertile parents. The survival rate from fertile age to post-fertile age is ω_2, and ω_3 denotes the probability that a post-fertile individual still lives (without the support by a fertile individual) when child care is needed. Now the fitness is $\lambda = \sqrt{\alpha_0 \omega_1}$.

3.1 Grandmother hypothesis

Now the question arises: When is the menopause adaptive? Consider the case when fertile individuals do not support grandmothers. We consider the following two cases: (i) Suppose that grandmothers do not help in child care, but their survival linearly reduces their own fecundity, i.e. $\omega_2(s) := s$ and $\alpha(s) := \alpha_0(1-s)$, where $s \in [0,1)$ is the cost spent on survival to post-fertile age (Fig. 3 depicts the situation). The fitness of the population is the long-term growth rate which can be calculated from the characteristic equation of the Leslie matrix:

$\lambda(s) = \sqrt{\alpha_0(1-s)\omega_1}$, and the optimal strategy is not to spend on own survival to post-fertile age. (ii) Suppose that grandmothers help in child care (Figure S 2.b). Let $\omega_2(s) := \overline{\omega}_2 s$, with some $\overline{\omega}_2$, $\alpha(s) := \alpha_0(1-s)$ and $\omega_1(s) := \omega_1 + a_{21}P(s)$, where $s \in [0,1)$, ω_1 is a „basic" survival rate, and the probability that a grandmother is alive when her help needed is $P(s) := \overline{\omega}_2 \omega_3 s$, i.e. we count only the help of those grandmothers who survive to the upper boundary of the third age class and do not count those who reach 'grandmother age' (reach the third class) but die before the upper boundary of age, and a_{21} denotes the efficiency of the grandmother's grandchild care. Clearly $a_{21}\overline{\omega}_2\omega_3 < 1$ and $\omega_1(s) = \overline{\omega}_1 + a_{21}\overline{\omega}_2\omega_3 s$, thus the fitness is

$$\lambda(s) = \sqrt{\alpha_0(1-s)(\overline{\omega}_1 + a_{21}\overline{\omega}_2\omega_3 s)}, \qquad \text{(SI 13)}$$

which is maximal at $s^* = \dfrac{a_{21}\overline{\omega}_2\omega_3 - \overline{\omega}_1}{2a_{21}\overline{\omega}_2\omega_3}$. Therefore, if the effect of grandchild care on the grandchild's survival is greater than his/her survival rate without this care, i.e. $a_{21}\overline{\omega}_2\omega_3 > \overline{\omega}_1$, then menopause is evolutionarily successful.

3.2 The Fifth Rule

Now the question arises: When is Fifth Rule adaptive? It requires us to support our elderly, which is possible only if the menopause has already become evolutionarily fixed, i.e. for fixed $s \in [0,1)$, let $\omega_2 := \overline{\omega}_2 s$ and $\alpha := \alpha_0(1-s)$. Let $y \in [0,1]$ denote the cost spent on the survival of post-fertile parents, and suppose that the negative effect of y on fecundity is linear: $\alpha(1-y)$, the children survival is $\omega_1(y) := \overline{\omega}_1 + aP(y)$, where $P(y) := \omega_2(\omega_3 + by)$ and b indicates how

efficiently the support to post-fertile parents by fertile individuals increases post-fertile survival, so $\omega_1(y) := \overline{\omega}_1 + a\omega_2(\omega_3 + by)$ (Fig. 4 depicts the situation). Now we have to maximize the fitness which can be calculated from the characteristic equation of the Leslie matrix, it is given by the following function in y:

$$\lambda(y) = \sqrt{\alpha(1-y)(\overline{\omega}_1 + a\omega_2(\omega_3 + by))}, \qquad \text{(SI 14)}$$

which attains its maximum at $y^* = \dfrac{a\omega_2(b-\omega_3) - \overline{\omega}_1}{2ab\omega_2}$. The latter is positive if $a\omega_2(b-\omega_3) - \overline{\omega}_1 > 0$. This condition is satisfied e.g., if the efficiency of the support to post-fertile parents is sufficiently large compared to the basic post-fertile survival rate.

3.3 A general multiplicative coevolution model

Now we set up a model combining the model of grandmother hypothesis and the model of the Fifth Rule. Our study will be based on two biological preconditions: First, since one can help a grandmother only if she is alive, for the development of the Fifth Rule, the existence of menopause is needed. Second, if a fertile mother gave away all her resources to help the survival of her mother, her fecundity would be zero. As before, let s be the cost a fertile female spends on her own survival to post-fertile age, and y the cost a fertile female spends on the survival of post-fertile parents. Based on the first precondition, unlike the additive approach of sections 3.2 and 3.3, we express the effect of strategies s and y on the demographic parameters in multiplicative form, considering the following strategy-dependent Leslie matrix:

$$L(s,y) := \begin{pmatrix} 0 & \alpha_0 \alpha(s,y) \\ \overline{\omega}_1 P(s,y) & 0 \end{pmatrix}, \qquad \text{(SI 15)}$$

where in both variables $P(s,y)$ is strictly monotonically increasing, and $\alpha(s,y)$ is strictly monotonically decreasing. Let us assume that strategies s and y act independently both on the fecundity and on the survival of children:

$$P(s,y) = p(s)q(y)\,,\ \alpha(s,y) = \beta(s)\gamma(y)\,, \qquad \text{(SI 16)}$$

where all one-variable functions are defined on [0,1].

Technical conditions on the functions involved:

a) p, q, β, γ are twice continuously differentiable.

b) $p(0) = q(0) = 1$, $\beta(0) = \gamma(0) = 1$. We note that these technical conditions imply that $\overline{\omega}_1$ and α_0 are the demographic parameters before the appearance of the considered traits, while $\beta(1) = \gamma(1) = 0$ expresses our second precondition.

c) $p'(s), q'(y) > 0$ $(s, y \in [0,1])$, $\overline{\omega}_1 p(1)q(1) < 1$.

d) $\beta'(s), \gamma'(y) < 0$ $(s, y \in (0,1])$, $\beta'(0) = \gamma'(0) = 0$.

Observe that conditions c) and d) are mathematical descriptions of trade-offs.

e) $p''(s), q''(y), \beta''(s), \gamma''(y) < 0$ $(s, y \in (0,1))$. (This condition will guarantee strict concavity of function z near its maximum).

Now, the fitness (unique positive eigenvalue of $L(s,y)$) is

$$\lambda(s,y) = \sqrt{\overline{\omega}_1 \alpha_0 p(s) q(y) \beta(s) \gamma(y)} \ (s, y \in [0,1]). \qquad \text{(SI 17)}$$

We will show that $\lambda(s,y)$ attains a strict local maximum at an interior point of the unit square $[0,1]\times[0,1]$. Indeed, maximization of $\lambda(s,y)$ is equivalent to the maximization of

$$z(s,y) = \overline{\omega}_1 \alpha_0 p(s) q(y) \beta(s) \gamma(y) \ (s, y \in [0,1]). \qquad \text{(SI 18)}$$

The first order necessary condition for the maximum attained at an interior point is

$$\frac{\partial}{\partial s} z(s,y) = q(y)\gamma(y)\big(\beta(s)p'(s) + p(s)\beta'(s)\big) = 0, \quad \text{(SI 19)}$$

$$\frac{\partial}{\partial y} z(s,y) = p(s)\beta(s)\big(\gamma(y)q'(y) + q(y)\gamma'(y)\big) = 0 \quad \text{(SI 20)}$$

Since p, q, β, γ are all positive in the interval (0,1), the above necessary condition is equivalent to

$$\varphi(s) := \beta(s)p'(s) + p(s)\beta'(s) = 0, \quad \text{(SI 21)}$$

$$\psi(y) = \gamma(y)q'(y) + q(y)\gamma'(y) = 0. \quad \text{(SI 22)}$$

From conditions b), c) and d) we obtain $\varphi(0) > 0$, $\varphi(1) < 0$, hence there is an $s^* \in (0,1)$ with $\varphi(s^*) = 0$. It is easy to check that conditions b), c), d) and e) also imply $\varphi'(s) < 0$, and hence φ is strictly decreasing, therefore s^* is its unique zero in the interval. $(0,1)$. Similar straightforward checking shows that $\psi(y)$ also has a unique zero y^* in the interval $(0,1)$. Hence (s^*, y^*) is a unique stationary point of function z in the interior of the unit square.

Now, for a second order sufficient condition for the maximum of function z, we calculate its Hessian:

$$H(s,y) = \begin{pmatrix} q(y)\gamma(y)\big(\beta(s)p''(s) + 2p'(s)\beta'(s) + p(s)\beta''(s)\big) & \big(\beta(s)p'(s) + p(s)\beta'(s)\big)\big(\gamma(y)q'(y) + q(y)\gamma'(y)\big) \\ \big(\beta(s)p'(s) + p(s)\beta'(s)\big)\big(\gamma(y)q'(y) + q(y)\gamma'(y)\big) & p(s)\beta(s)\big(\gamma(y)q''(y) + 2q'(y)\gamma'(y) + q(y)\gamma''(y)\big) \end{pmatrix},$$

(SI 23)

Observe that from $\varphi(s^*) = 0$ and $\psi(y^*) = 0$, we obtain

$$H(s^*, y^*) = \begin{pmatrix} q(y^*)\gamma(y^*)\big(\beta(s^*)p''(s^*) + 2p'(s^*)\beta'(s^*) + p(s^*)\beta''(s^*)\big) & 0 \\ 0 & p(s^*)\beta(s^*)\big(\gamma(y^*)q''(y^*) + 2q'(y^*)\gamma'(y^*) + q(y^*)\gamma''(y^*)\big) \end{pmatrix}$$

From conditions a)-e), we easily get

$$q(y^*)\gamma(y^*)\big(\beta(s^*)p''(s^*) + 2p'(s^*)\beta'(s^*) + p(s^*)\beta''(s^*)\big) < 0 \qquad \text{(SI 24)}$$

$$p(s^*)\beta(s^*)\left(\gamma(y^*)q''(y^*) + 2q'(y^*)\gamma'(y^*) + q(y^*)\gamma''(y^*)\right) < 0, \qquad \text{(SI 25)}$$

implyig $\mathrm{tr}H(s^*,y^*) < 0$ and $\det H(s^*,y^*) > 0$, i.e. $H(s^*,y^*)$ is negative definite. Therefore (s^*,y^*) is a strict local maximum point. Since (s^*,y^*) is the unique stationary point, it is also a strict global maximum point in the interior of the unit square.

Finally, we note that, if Hessian $H(s,y)$ is negative definite in the interior of the unit square, then function λ is globally strictly concave, and therefore (s^*,y^*) is a global maximum point of λ. In the terminology of fitness landscapes, in the sense of any reasonable strategy dynamics the species will evolve into the evolutionarily optimal behavior (s^*,y^*).

3.4 Numerical Examples

In this section, by numerical study, we illustrate the effect of different (linear, convex and concave) trade-offs on the level of the optimal backward help (*y**). We calculated the maximal long-term growth rate (fitness) of various populations as a function of y from the characteristic equation of the corresponding Leslie matrix. The value of y that gives the highest long-term growth rate termed as the optimal backward help (*y**). We also calculated the number of offspring and the offspring survival given the optimal *y**. We investigated the effects of different cost-benefit parameters on the evolvability of backward help (*y*). Life-history parameters are based on the figures from Mace [1]· It is possible to generate all the possible combinations of cost-benefit trade-offs by setting the appropriate cost, benefit parameters to zero (*c*, *d*, *h*). Also, convex or concave cost-benefit functions can be achieved by setting the appropriate parameters (*c*, *d*, *h*) to smaller or to greater than one (see Table S1 for a summary of parameters). We used the following general Leslie matrix (see Fig. 5 for a schematic description):

$$\begin{pmatrix} 0 & \alpha(1-y)^{c}+a_{12}\,\omega_2(1-y)^{d}\left(\omega_3+b\left(1-(1-y)^{h}\right)\right) \\ \varpi_1+a_{21}\,\omega_2(1-y)^{d}\left(\omega_3+b\left(1-(1-y)^{h}\right)\right) & 0 \end{pmatrix}$$

Table S1. Parameters of the model.

Life-history parameters

α: Number of offspring
ω_1: survival of the first age class (offspring)
ω_2: survival of the first reproductive class (parents)
ω_3: survival of the non-reproductive class (grandparents)

Benefit-parameters

a_{12}: efficacy of the granny's help on the fecundity of the parent
a_{21}: efficacy of the granny's help on the survival of the offspring
b: effectiveness of IT, the maximum efficacy of the parents help on the grandparent's survival h: efficacy of the parents help (steepness)

Cost parameters

c: cost of helping grannies on the fecundity of the parent (steepness)
d: cost of helping grannies on the survival of the parent (steepness)

Four possible combinations exist in terms of the benefit functions: (i) a_{12}, $a_{21} > 0$; (ii) $a_{12} > 0$, $a_{21} = 0$, (iii) $a_{12} = 0$, $a_{21} > 0$; and (iv) a_{12}, $a_{21} = 0$. In the first case, grandmothers give benefits for both the number of offspring and for the survival of them, in the second case they give benefit only for the number of offspring; in the third case they only give benefit for the survival of the offspring and finally, in the last case, they do not provide any benefit. This last case is not interesting for us, thus it will not be investigated any further.

In the same way, four possible combinations exist in terms of the cost functions: (i) c, $d > 0$; (ii) $c > 0$, $d = 0$, (iii) $c = 0$, $d > 0$; and (iv) c, $d = 0$. In the first case helping grandmothers imposes a cost on both the parents' reproductive output and on the parents' survival, in the second case only on the number of offspring, in the third only on the survival of the parent, and finally, in the last case it imposes no cost at all. Just as before, this last case

is not interesting for us, thus it will not be investigated any further. See Table S2 for investigated parameter combinations.

Table S2. The investigated parameter combinations (see Figures S1-S7 for the corresponding results).

	Grandparental help	Shape of the cost function	Shape of the benefit function	Figure:
1.	a_{12}, $a_{21} > 0$	$d = 1$	$h = 1$	S1
2.		$d = 1$	$h = 2$	S2
3.		$d = 0.5$	$h = 2$	S3
4.	$a_{12}= 0$, $a_{21} > 0$	$d = 1$	$h = 1$	S4
5.		$d = 0.5$	$h = 2$	S5
6.	$a_{12}> 0$, $a_{21} = 0$	$d = 1$	$h = 1$	S6
7.		$d = 0.5$	$h = 2$	S7

3.5 Illustrative numerical examples: results

IT evolves the most readily when the grandparental help increases both the survival of the offspring and the number of offspring (Figure S1-S3). Linear cost and benefit functions do not favour the evolution of IT (Figs. S1, S4, S6, d=1, h=1); conversely, convex benefit and concave cost functions promote the evolution of IT (Fig. S2, S3, S5, S7, d=0.5, h=2). It is possible to find cost parameters (c, d) where IT evolves even if the efficacy parental transfer and grandparental help (a_{21} and b respectively) is low (Figs. S2, S3). Conversely, it is possible

to find (high) a_{21}, b parameters where IT evolves even if it imposes a high cost on the survival of the parents or on the number of offspring (d and c, respectively, see Figs. S1, S2).

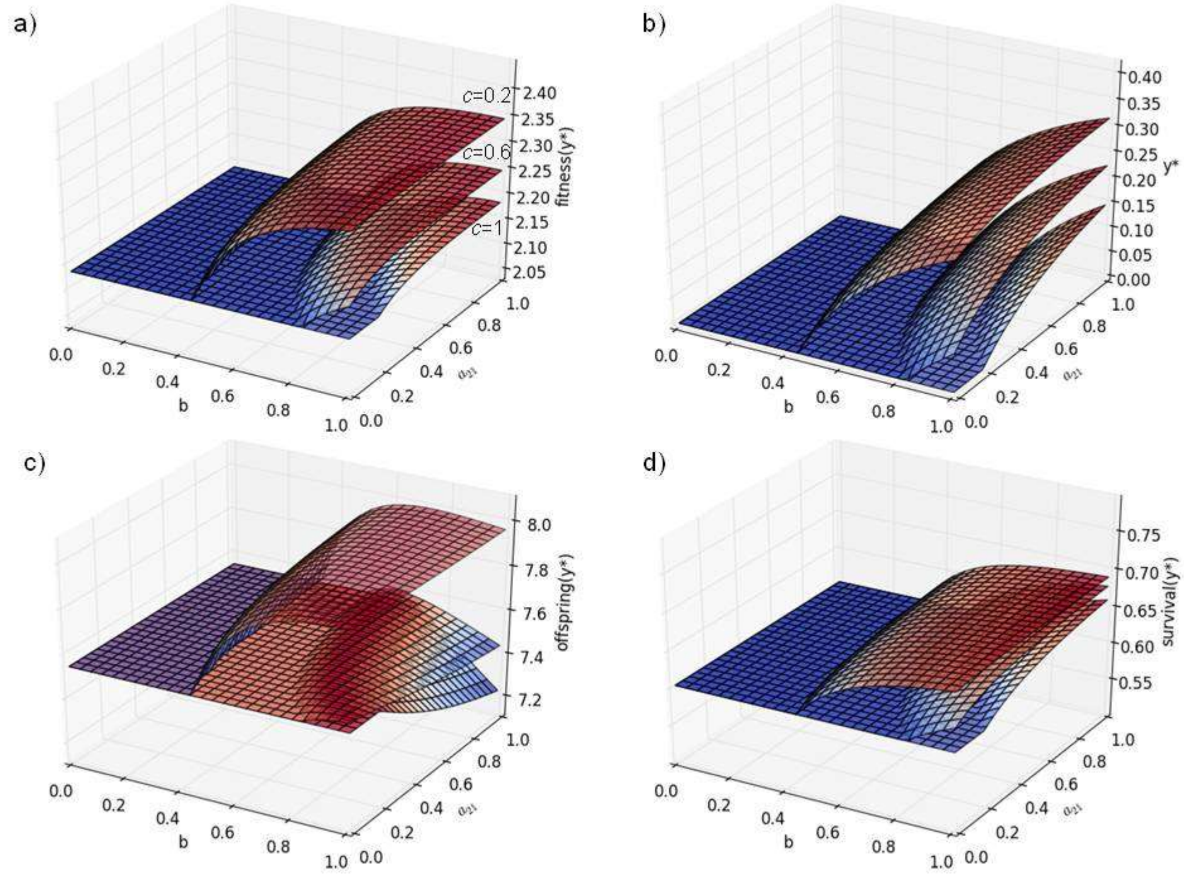

Figure S1. Numerical example for the Fifth Rule, grandmothers increase both offspring survival and the number of offspring (a_{12}, $a_{21} > 0$). a) Dominant eigenvalues (fitness); b) corresponding y^* value; c) offspring number as a function of y^*; d) survival of offspring as a function of y^*; all four subfigures plotted as a function of the effectiveness of IT (b) and the efficacy of the grandparental help on the survival of the offspring (a_{21}). Parameters: α_2=6, ω_1=0.45, ω_2=0.62, ω_3=0.25, d = 1, h = 1, c =0.2, 0.6, 1; a_{12}=10.

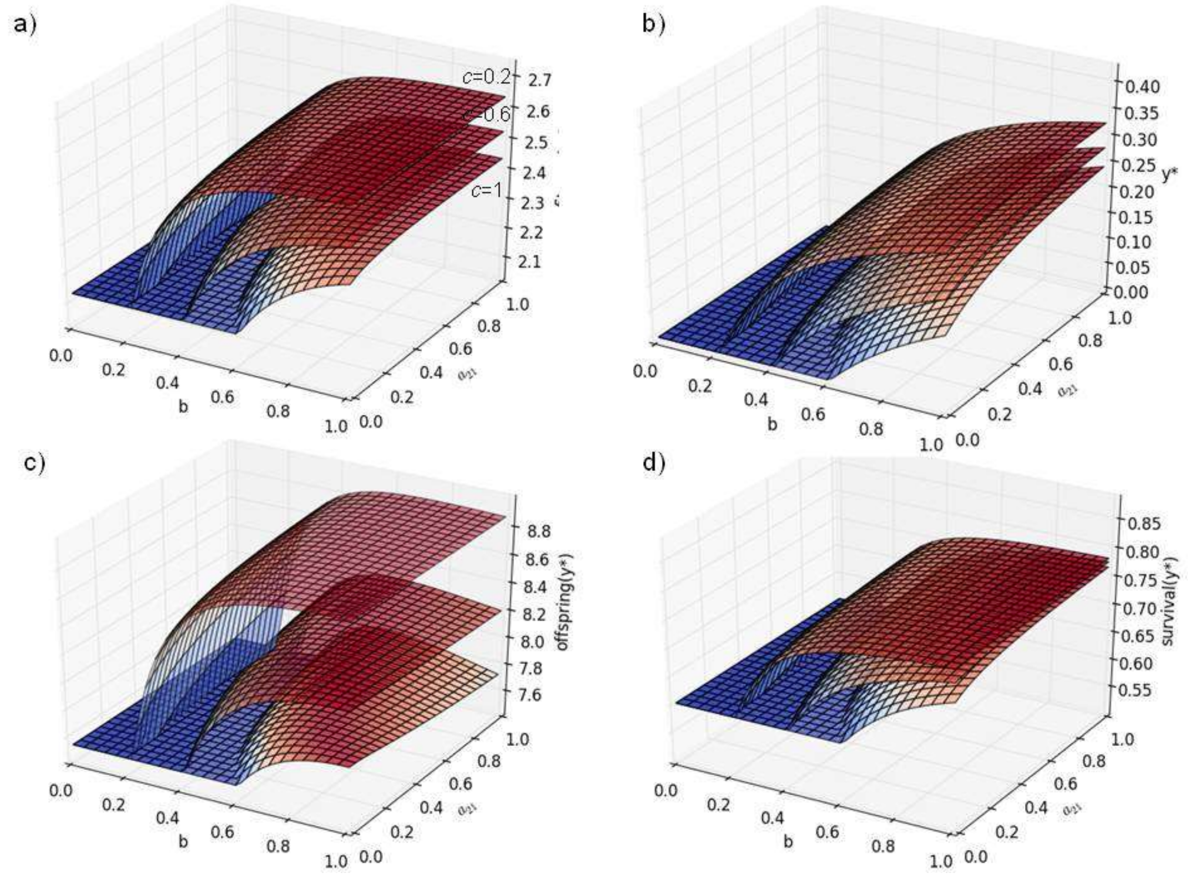

Figure S2. Numerical example for the Fifth Rule: grandmothers increase both offspring survival and the number of offspring (a_{12}, $a_{21} > 0$). a) Dominant eigenvalues (fitness); b) corresponding y^* value; c) offspring number as a function of y^*; d) survival of offspring as a function of y^*; all four subfigures plotted as a function of the effectiveness of IT (b) and the efficacy of the grandparental help on the survival of the offspring (a_{21}). Parameters: α_2=6, ω_1=0.45, ω_2=0.62, ω_3=0.25, d = 1, h = 2, c =0.2, 0.6, 1; a_{12}=10.

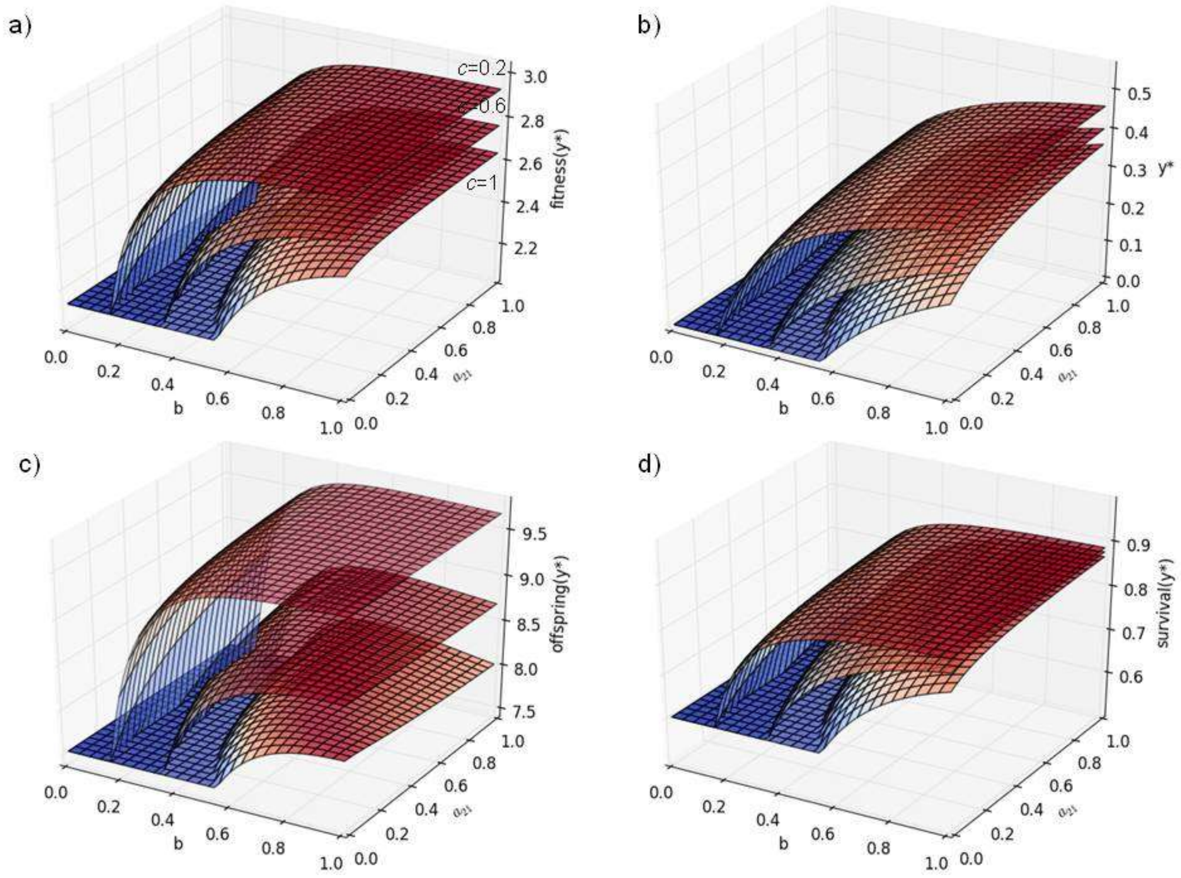

Figure S3. Numerical example for the Fifth Rule: grandmothers increase both offspring survival and the number of offspring (a_{12}, $a_{21} > 0$). a) Dominant eigenvalues (fitness); b) corresponding y^* value; c) offspring number as a function of y^*; d) survival of offspring as a function of y^*; all four subfigures plotted as a function of the effectiveness of IT (b) and the efficacy of the grandparental help on the survival of the offspring (a_{21}). Parameters: α_2=6, ω_1=0.45, ω_2=0.62, ω_3=0.25, d = 0.5, h = 2, c =0.2, 0.6, 1; a_{12}=10.

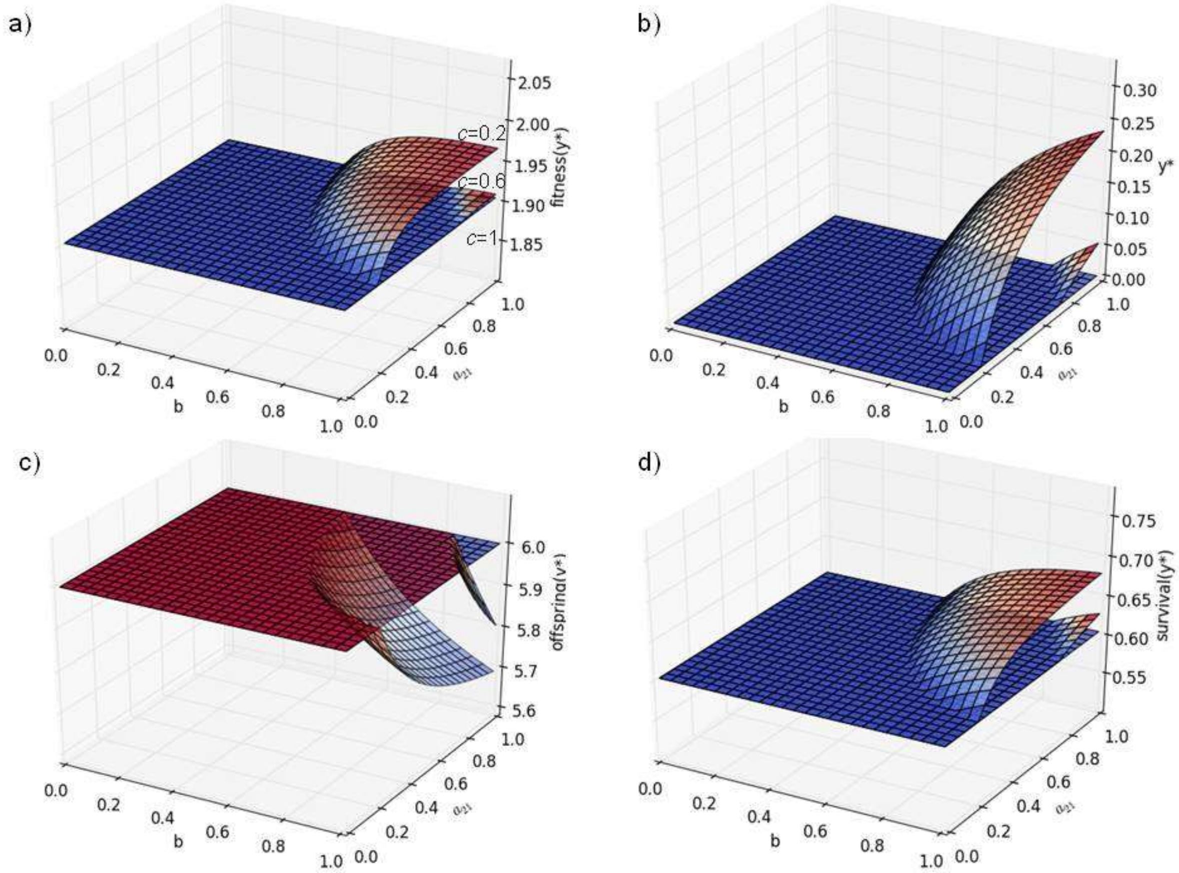

Figure S4. Numerical example for the Fifth Rule, grandmothers only increase offspring survival (a_{12}= 0, a_{21} > 0). a) Dominant eigenvalues (fitness); b) corresponding $y*$ value; c) offspring number as a function of $y*$; d) survival of offspring as a function of $y*$; all four subfigures plotted as a function of the effectiveness of IT (b) and the efficacy of the grandparental help on the survival of the offspring (a_{21}). Parameters: α_2=6, ω_1=0.45, ω_2=0.62, ω_3=0.25, d = 1, h = 1, c =0.2, 0.6, 1; a_{12} =0.

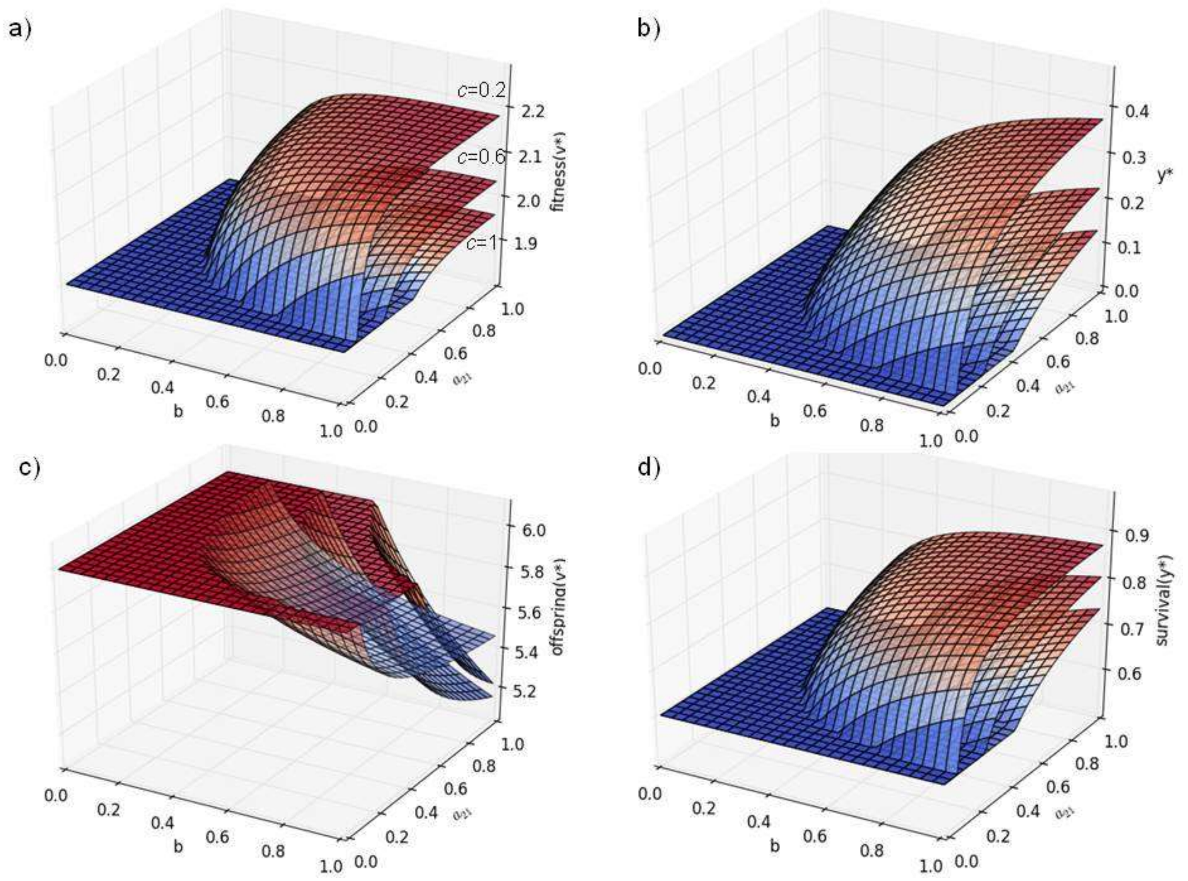

Figure S5. Numerical example for the Fifth Rule, grandmothers only increase offspring survival (a_{12}= 0, a_{21} > 0). a) Dominant eigenvalues (fitness); b) corresponding y^* value; c) offspring number as a function of y^*; d) survival of offspring as a function of y^*; all four subfigures plotted as a function of the effectiveness of IT (b) and the efficacy of the grandparental help on the survival of the offspring (a_{21}). Parameters: α_2=6, ω_1=0.45, ω_2=0.62, ω_3=0.25, d = 0.5, h = 2, c =0.2, 0.6, 1; a_{12} =0.

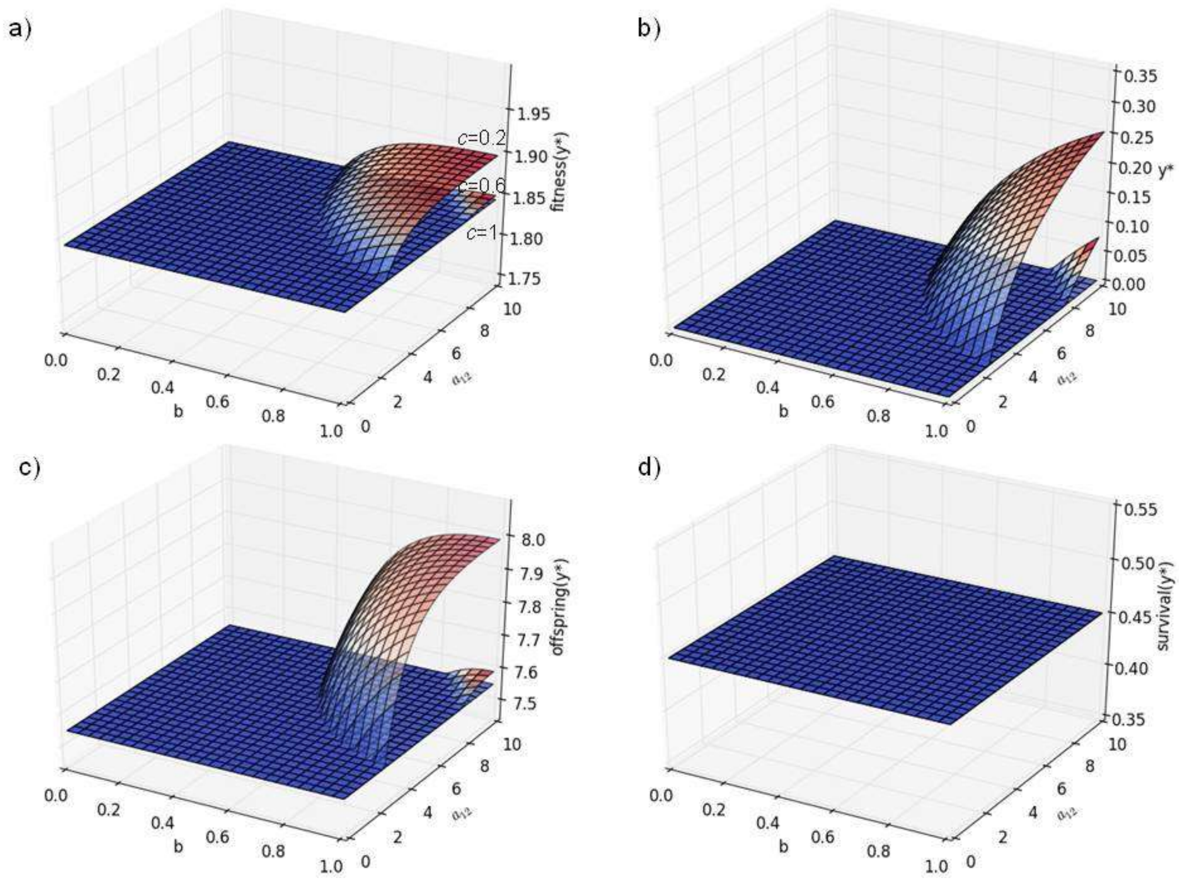

Figure S6. Numerical example for the Fifth Rule, grandmothers only increase offspring number (a_{12}> 0, a_{21} = 0). a) Dominant eigenvalues (fitness); b) corresponding y* value; c) offspring number as a function of y*; d) survival of offspring as a function of y*; all four subfigures plotted as a function of the effectiveness of IT (b) and the efficacy of the grandparental help on the fecundity of the parent (a_{12}). Parameters: α_2=6, ω_1=0.45, ω_2=0.62, ω_3=0.25, d = 1, h = 1, c =0.2, 0.6, 1; a_{21} = 0.

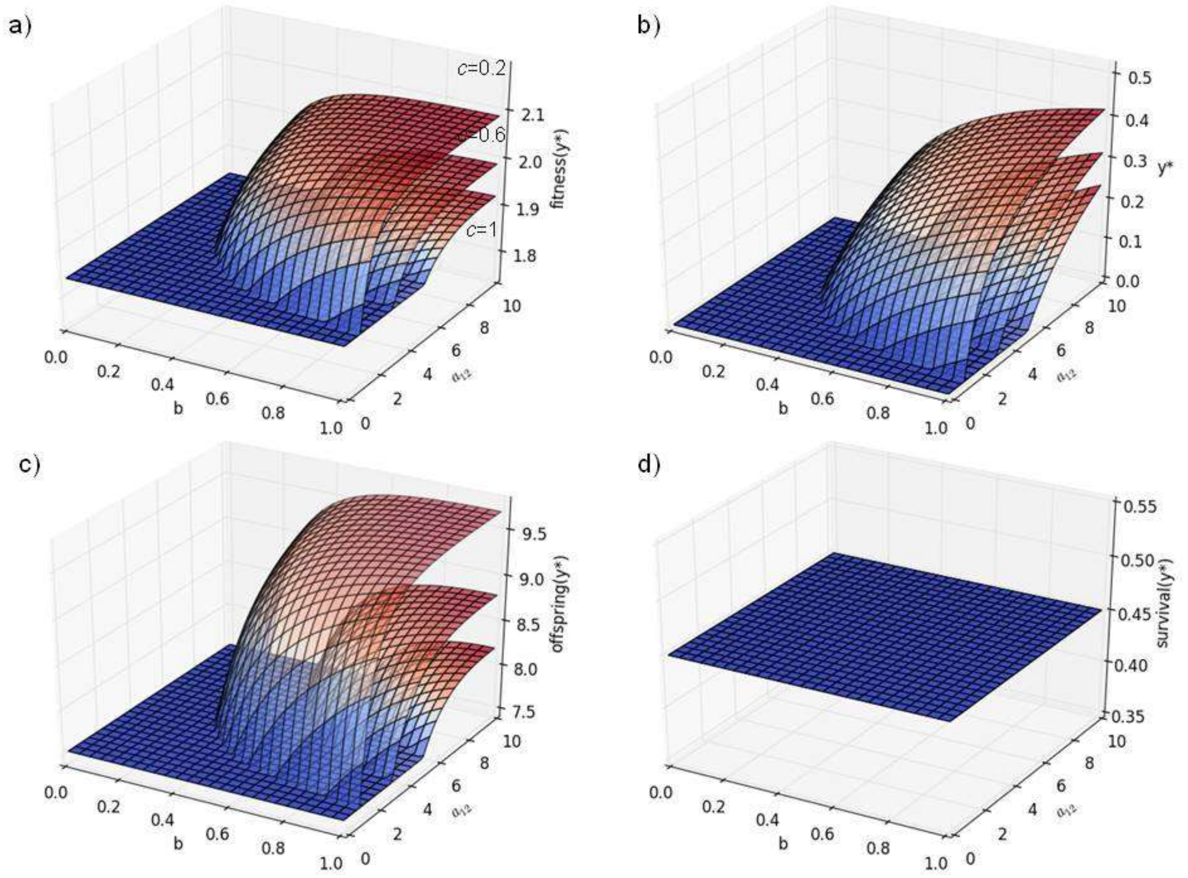

Figure S7. Numerical example for the Fifth Rule, grandmothers only increase offspring number (a_{12}> 0, a_{21} = 0). a) Dominant eigenvalues (fitness); b) corresponding y* value; c) offspring number as a function of y*; d) survival of offspring as a function of y*; all four subfigures plotted as a function of the effectiveness of IT (b) and the efficacy of the grandparental help on the fecundity of the parent (a_{12}). Parameters: α_2=6, ω_1=0.45, ω_2=0.62, ω_3=0.25, d = 0.5, h = 2, c =0.2, 0.6, 1; a_{21} = 0.

References

1. Mace R: **Evolutionary ecology of human life history**. *Animal behaviour* 2000, **59**(1):1-10.
